# Letter from Our Correspondent at the American Medical Association

**Published:** 1882-07-20

**Authors:** T. B. Greenly

**Affiliations:** Ord, Kentucky


### CORRESPONDENCE.

### A LETTER FROM OUR CORRESPONDENT AT THE AMERICAN MEDICAL ASSOCIATION.

*Editors Southern Medical Record:*

Being appointed by our State Society a delegate to the American Medical Association at its meeting at Saint Paul, I arrived there on Tuesday morning in time to register before the 11 o'clock a. m. session.

Owing to the absence of the President, Dr. Woodward, who is in Europe on account of health, the Annual Address was delivered by Dr. Hooper, of Arkansas, First Vice-President. As far as I heard an opinion expressed, it was universally admitted to be, not only pertinent to the occasion, but masterly in elocution and beautiful in diction; and I think, with all the learning and research of our worthy President, the occa ion could not have been made more interesting or entertaining had he been present.

Doctor Hooper, our excellent *pro tem*, is a gentleman of fine presence, portly and dignified, and seemed to be at perfect ease under all the dignities of his office. He presided with a great deal of courtesy, and seemed to be just in all of his decisions. He might, with propriety, be classed as a model Southern gentleman.

There was an unusually large attendance at Saint Paul—only falling short a very few of one thousand delegates—I think 980 being registered the third day.

This large number being present in that comparatively sparcely settled country, perhaps, may be accounted for to some extent, because the Northern railroads offered such great inducements for the members to travel in the great Northwest. Then, again, possibly, many had never been to Saint Paul, and availed themselves of that pleasure, as well as to attend the meeting of the Association.

I can assure you, in speaking for myself, that a visit to the Northwest affords no little pleasure at this season of the year, to say nothing of being present at our meeting.

The morning sessions were well attended, the hall being generally crowded; many ladies availing themselves of the opportunity of seeing a big meeting of doctors.

The credentials of the delegation of the New York State Society were referred to the Judicial Council, which reported that the

New Code of Ethics adopted by said Society at its last meeting was so diametrically in opposition to that of the Association that said delegation could not be received. This decision of the Judicial Council was received by the Association with great applause. It would not be surprising if the physicians of New York, at the next annual meeting of their Society, rallied in sufficient numbers to reconsider the work of its last meeting, and as far as the amendment or change of the Code of Ethics is concerned, abrogate it entirely. The adoption of that change was only effected by a very small majority of a very small portion of membership. I think there were only about 76 members present, and the amendment to the Code, allowing consultation with Homeopaths, was carried by some 39 or 40 votes. It will be observed that this was a very meagre majority of a very small number present of so numerous a body as the Medical Society of the State of New York. It is charged that Dr. St. John Roosa, of New York city, introduced the amendment in the interest of the specialists of the city. This I can hardly believe of Dr. Roosa, as I regard him as being entirely above any such selfish motives. As far as my limited acquaintance with him extends, I regard him both as a gentleman and honorable man; and have no doubt he was sincere and honest in his resolutions respecting the Code of Ethics.

But if we are honest, the question might be asked how can we consult with our Homeopathic friends? I am somewhat at a loss in being able to formulate an answer to this question. In fact, if both sides are honest, I cannot see how it is possible for us to consult. What is there to consult about?

If the patient is sick enough for either side to want counsel, he needs medical treatment, and as our opponents don't profess to give anything but Hahnemann's decillionth doses, an understanding between us could not be arrived at.

Therefore, unless we could convince them of the necessity of medication, there would be no understanding or agreement—consequently, as before remarked, nothing to consult about. Were we to succeed in convincing them that our plan was essential, then their theory would be destroyed and their occupation gone. But, remarks some one, there is a split among them, some still adhering strictly to the Hahnemannic theory, while others are practicing pretty much as we do. The first are termed the high school, or you might say Bourbons. They are the straight-laced, high-toned, aristocratic Homeopaths. The others claim to be more liberal, and where purely homeopathic remedies are not indicated, or fail to do good, they assume the privilege of using what they term Allopathic Medicines. Although, as a rule, we should be in favor of advancement in any department of science, yet we should contend for honesty at the same time. Therefore the Bourbons of homeopathy, I think, are more entitled to our respect for practicing the principles they profess, than those who claim to be followers of Hahnemann but practice to the contrary. It strikes us very plainly that if a man sets himself up as a Homeopath and practices as a regular, he is practicing a fraud on the credulity of the people.

Doctor Dennison, of Colorado, introduced a resolution to the effect that as the regular profession are called Allopaths by the Homeopathists, and the laity, in many instances, entertaining the same view, of course ignorantly, the Association should take some steps to declare our proper status, so that all would know that we belong to no particular school, or practice no special dogma, but obtain our remedies from all sources and kingdoms—vegetable, mineral and animal alike, and reject nothing by which good can be derived in the treatment of disease. This resolution was referred to the Judicial Council, which reported that it did not come under its jurisdiction.

When it came up for discussion it was tabled by a small majority, on the ground that our profession was so well established in principles that it needed no resolutions explanatory, and that such resolutions would only tend to magnify the matter in the estimation of our opponents. A resolution of this kind was afterwards adopted in a modified form.

Take the meeting as a whole, I think it will compare favorably in the way of scientific work with a majority of our previous assemblies.

The reports from the various sections were quite able, and evinced a great deal of research.

In the sections, many papers of great interest were read and discussed.

I had the pleasure of meeting a few of the *big* men of your State, prominently among whom were Drs. Campbell and Batty. They both took quite an active part in the discussion of gynecological matters in the section on obstetrics, and Dr. Batty gave us a very able impromptu report of his observations in Europe last year. He was a delegate to the International Congress at London. This report pertained mostly to the progress making in his special department. He was not only interesting but quite amusing.

I had hoped to meet your eminent citizen, Dr. T. S. Powell, whom I had the pleasure of becoming acquainted with at Richmond last year.

The Association never enjoyed a more pleasant meeting than at Saint Paul. Our brethren of that city and State of Minnesota vied with each other how hospitably they could treat us. We were entertained on a scale both magnificent and munificent. Although we surprised them with numbers, the committee of arrangements proved themselves equal to the emergency, and made us all comfortable and happy.

It would be impossible to speak in too high terms of the courtesy of Dr. Stone, chairman of the committee, and the onerous and almost incessant work he performed for our accommodation and welfare. He is an energetic, whole-souled gentleman.

After adjournment on Friday afternoon, many of the members left on a Northwestern tour, all having passes on the various Northwestern railroads. Your humble servant visited Lake Superior, passed west through Minnesota, Dakota and into Montana on the Yellow Stone as high as Miles City, being 500 miles west of Fargo on Red River. Thence on our return we visited the city of Winnepeg, in Manitoba, 63 miles north of the British line. This city is in the 50th degree of north latitude. The weather, during all this trip, was fine, and I did not use my overcoat on account of cold. We passed through a great deal of fine agricultural and grazing country.

On inquiry of the Doctors, at most of the towns I visited, I learned the more prevalent diseases were rheumatism, neuralgia, typhoid fever, and in the spring some lung troubles. This was the case in Fargo, Bismarck, Miles City, Mondar and Winnepeg. I attribute the prevalence of typhoid fever in those towns mainly to the want of proper drainage.

Everything in that vast country is on such a boom that the people have but little time to devote to sanitary matters. The country is full of people waiting for houses, and so they get a house to shelter in, whether it is built over a mud-hole or not, they seem to be satisfied.

They have very severe winters up there, but the atmosphere is so very dry, they say the cold is not very disagreeable. The mercury frequently gets as low as 40° below zero.

*Ord, Kentucky, June, 1882.* T. B. GREENLY, M. D.

---

A QUACK DOCTOR, on his death-bed, willed his property to a lunatic asylum, giving as a reason for so doing that he wished his fortune to go to the liberal class who had patronized him.

